# PD29 - Do we have a specialist allergy service?

**DOI:** 10.1186/2045-7022-4-S1-P29

**Published:** 2014-02-28

**Authors:** Nicola Jay, Fiona Shackley, Catherine Waruiru

**Affiliations:** 1 Sheffield Children’s Hospital, Sheffield, United Kingdom

## 

Allergy Services in the UK are diverse in terms of place of delivery and by whom. With current re-organization of the NHS there is a need to provide more clear definition of what constitutes a tertiary allergy service. In an attempt to clarify our own position, we undertook a prospective evaluation of all paediatric allergy clinic attendees at Sheffield Children's Hospital during November 2012.

The **aim** being to identify the primary diagnosis and any cofactors that would affect that condition.

## Results

Data ascertainment occurred in 93 patients of whom 29 were new patients. IgE mediated food allergy was present in 53 children with 17 allergic to more than one food, egg allergy being most prevalent. Of these, 34 had associated atopy with 31 eczema, 21 asthma and 13 hay fever. Only 13 of the single food reactions didn’t have associated atopy. Ten children were thought to have non IgE mediated disease with equal spread of skin and gastro-intestinal symptoms. Seven of the new patients had been referred due to concerns over drug allergy and 14 had a diagnosis of urticarial/angioedema (4 chronic, 3 intermittent and 7 acute). Seven children had allergic rhinitis with co-sensitisation to a number of aeroallergens. There was one case each of mastocytoma, wasp allergy and eosinophilic oesophagitis. Only 5 children were thought to be avoiding food without any good reason.

## Discussion

Our review demonstrates the significant atopic burden of children attending our clinic. 75% of patients had complex disease (IgE food, non IgE food, chronic urticaria, drug allergy, rhinitis, other) that requires a multiprofessional approach which, at the moment, only a allergy clinic can provide (dietician, spirometry, co-located specialities). What is tertiary and secondary level allegy is more difficult to define.

